# Long-Term Results of Hybrid Left Ventricular Reconstruction in the Treatment of Ischemic Cardiomyopathy

**DOI:** 10.1007/s12265-021-10133-9

**Published:** 2021-05-11

**Authors:** Jan Naar, Ivo Skalský, Andreas Krűger, Filip Málek, Kevin Van Bladel, Lon S. Annest, Petr Moučka, Tomáš Mráz, Vivek Y. Reddy, Petr Neužil

**Affiliations:** 1 Department of Cardiology, Na Homolce Hospital, Röentgenova 37/2, 150 30 Prague 5, Czech Republic; 2 Department of Cardiac Surgery, Na Homolce Hospital, Prague, Czech Republic; 3 BioVentrix, Inc., San Ramon, CA USA; 4 Cardiac Arrhythmia Service, Mount Sinai Medical Center, New York, USA

**Keywords:** Hybrid approach, Ischemic cardiomyopathy, Left ventricular aneurysm, Left ventricular reconstruction

## Abstract

The evidence supporting surgical aneurysmectomy in ischemic heart failure is inconsistent. The aim of the study was to describe long-term effect of minimally invasive hybrid transcatheter and minithoracotomy left ventricular (LV) reconstruction in patients with ischemic cardiomyopathy. Twenty-three subjects with transmural anterior wall scarring, LV ejection fraction 15–45%, and New York Heart Association class ≥ II were intervened using Revivent TC anchoring system. LV end-systolic volume index was reduced from 73.2 ± 27 ml at baseline to 51.5 ± 22 ml after 6 months (*p* < 0.001), 49.9 ± 20 ml after 2 years (*p* < 0.001), and 56.1 ± 16 ml after 5 years (*p* = 0.047). NYHA class improved significantly at 5 years compared to baseline. Six-min walk test distance increased at 2 years compared to the 6-month visit. Hybrid LV reconstruction using the anchoring system provides significant and durable LV volume reduction during 5-year follow-up in preselected patients with ischemic heart failure.

**Graphical abstract:**

**Figure:**
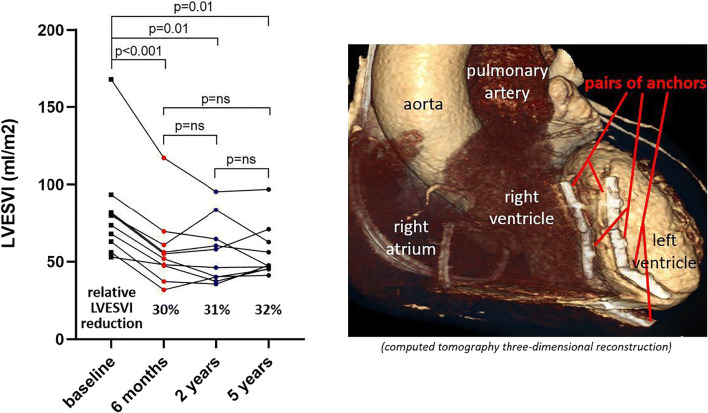
*Legend*: Hybrid left ventricular reconstruction using the anchoring system provides significant and durable LV volume reduction throughout 5-year follow-up in preselected patients with ischemic heart failure.

**Supplementary Information:**

The online version contains supplementary material available at 10.1007/s12265-021-10133-9.

## Introduction

Heart failure (HF) represents a substantial healthcare issue in developed countries [1, 2], and coronary heart disease is a major cause of HF with reduced ejection fraction (EF) in the western world [3]. Ischemic HF is associated with shorter survival than non-ischemic HF [4, 5], but conventional pharmacological HF therapy is usually not able to be addressed to a specific etiology. Thus, there is a potential for new treatment strategies that could diminish increased mortality and morbidity in patients with ischemic cardiomyopathy.

Exclusion of the scarred, akinetic, or dyskinetic tissue from the left ventricle (LV) might be beneficial in patients that have experienced extensive transmural myocardial infarction in the region of the left anterior descending artery. Remodeling procedure leads to volume reduction and geometry restoration of the dilated LV that secondarily results in decreased wall tension and lower myocardium energy demand [6, 7].

LV reconstruction in ischemic cardiomyopathy has generally been performed in the setting of standard open-heart surgery, where aneurysm resection is usually accompanied by other interventions, mostly coronary artery revascularization [8–12]. Hybrid transcatheter and minithoracotomy LV reconstruction represents a novel treatment option for this cohort of patients with favorable safety and efficacy *short-term* results [13–15]. It is a remodeling procedure offering minimally invasive access without the need of sternotomy and cardiopulmonary bypass. Higher preoperative LV end-systolic volume index (LVESVI) seems to be an adverse outcome of surgical aneurysmectomy [16, 17] even minimally invasive reconstruction [14].

## Methods

### Study Population

Eligible subjects with ischemic LV dysfunction due to prior myocardial infarction leading to transmural scarring with akinesis or dyskinesis in the anteroseptal, apical, or apicolateral region were enrolled at our center between September 2013 and March 2019. The main inclusion criteria were age 18–80 years, LV EF 15–45%, New York Heart Association (NYHA) class II–IV, stable HF medication for > 90 days, and willingness and ability to comply with the study procedures. Localization, size, and transmural extent of the scar were objectified by cardiac magnetic resonance imaging or dynamic computed tomography prior to enrollment. The main exclusion criteria were intracardiac thrombus, myocardial infarction within 90 days before the procedure, systolic pulmonary arterial pressure > 60 mmHg assessed by echocardiography, previous left-sided thoracotomy, and contraindication to open-heart surgery (in case of a complication).

### Study Protocol

The study was designed as prospective and single arm, evaluating 5-year follow-up data. The primary efficacy endpoint was the reduction of LVESVI. Secondary efficacy endpoints were changes in NYHA class, 6-min walk test (6-MWT), Minnesota Living with Heart Failure Questionnaire (MLHFQ), and a level of N-terminal prohormone of brain natriuretic peptide (NT-proBNP). Transthoracic echocardiography, NYHA class, 6-MWT, MLHFQ, and NT-proBNP were obtained at baseline and at 6-month, 2-year, and 5-year follow-up visits. NYHA class and 6-MWT were assessed by different physicians (trained persons), who were blinded to results from preceding follow-up visits. Echo data were evaluated by experienced cardiologist, who was blinded to echo results from other follow-up visits and to timing of echo control. LV volumes and EF were assessed offline on two-dimensional echocardiography images with Q-Station software, version 3.8.5 (Philips Healthcare, Andover, USA), using Simpson’s biplane method [18]. The study protocol was approved by the institutional ethics committee and complied with the principles outlined in the Declaration of Helsinki. All participants provided written informed consent to participate in the study.

### Procedure

LV reconstruction was performed under general anesthesia as a stand-alone hybrid transcatheter and minimally invasive surgical remodeling procedure on the beating heart utilizing the Revivent TC system (BioVentrix, Inc., San Ramon, USA). As described in detail previously [13, 14], the system consists of delivery equipment and implantable components. The implantable components are created from pairs of titanium anchors covered in polyester cloth, connected by a poly-ether-ether-ketone tether. A hinged anchor is delivered by internal jugular vein access from the right side of the interventricular septum and the locking anchor by left-sided minithoracotomy. The reconstruction is often completed by exclusively LV-LV placement of the anchor pair on the LV apex. Oral anticoagulation therapy with warfarin was initiated after the procedure and maintained at an international normalized ratio 2.0–2.5 for the period of at least 3 months.

### Statistical Analysis

To evaluate changes in variables over time, a repeated-measures one-way ANOVA using Tukey’s multiple comparisons test was used for analysis of complete data and a mixed-effects model using REML (residual maximum likelihood) method with fixed effect type III was applied for the analysis with missing data. An unpaired two-tailed *t*-test or an unpaired two-tailed *t*-test with Welch’s correction (where the equal standard deviation was not assumed) were used to evaluate the association between baseline LVESVI and changes in clinical variables. Data are presented as mean ± standard deviation. Statistical analyses were performed using GraphPad Prism, version 8.2.1 (GraphPad Software, Inc., La Jolla, CA, USA); *p*-values < 0.05 were considered statistically significant.

## Results

Twenty-three patients (15 male, mean age 59 ± 11 years) were recruited. Mean LV EF was 32 ± 7%, mean LVESVI 75 ± 25 ml/m^2^, and mean NYHA class 2.3 ± 0.5. Mean follow-up time was 5.1 years (minimum 1.9 years, maximum 7.4 years). Twenty patients completed a 6-month visit, 18 patients a 2-year visit, and 11 patients a 5-year visit. Table 1 details the baseline characteristics of the study participants. Table 2 shows the differences in HF pharmacotherapy at baseline compared to the end of the follow-up.

**Table 1:** Preoperative baseline characteristics

Variable	*n* = 23
Age (years)	59 ± 11
Male sex (*n*; %)	15 (65%)
BMI (kg/m^2^)	29 ± 6
Systolic blood pressure (mmHg)	137 ± 19
Diastolic blood pressure (mmHg)	79 ± 11
NYHA class	2.3 ± 0.5
LVEF (echo, %)	32 ± 7
6-MWT (m)	381 ± 103
MLHFQ (points)	22
LVEDVI (echo; ml/m^2^)	107 ± 27
LVESVI (echo; ml/m^2^)	75 ± 25
Mitral regurgitation (0–4)	1.2
Previous CABG/PCI (*n*; %)	15 (65%)
ICD implanted (*n*; %)	19 (83%)
CRT implanted (*n*; %)	3 (13%)
Arterial hypertension (*n*; %)	14 (61%)
Diabetes mellitus (*n*; %)	5 (22%)
Smoker: current/past/never (*n*; %)	3/17/3 (13/74/60%)
% of patients on ß-blocker/ACEI or ARB/MRA	96/91/65
% of target daily dose of ß-blocker	37
% of target daily dose of ACEI/ARB	50

*ACEI*, angiotensin-converting enzyme inhibitor; *ARB*, angiotensin II receptor blocker; *BMI*, body mass index; *CABG*, coronary artery bypass grafting; *CRT*, cardiac resynchronization therapy; *ICD*, implantable cardioverter defibrillator; *LVEDVI*, left ventricular end-diastolic volume index; *LVEF*, left ventricular ejection fraction; *LVESVI*, left ventricular end-systolic volume index; *MLHFQ*, Minnesota Living with Herat Failure Questionnaire; *MRA*, mineralocorticoid receptor antagonist; *NYHA*, New York Heart Association; *PCI*, percutaneous coronary intervention; *6-MWT*, 6-min walk test

**Table 2:** Changes in heart failure medical therapy during follow-up

	Baseline (*n* = 19)	End of follow-up (*n* = 19)
% of target daily dose of ß-blocker	39	27
% of target daily dose of ACEI/ARB	51	42
% of patients on MRA	63	74
% of patients on sacubitril/valsartan	11	26

Differences in background heart failure pharmacotherapy of subjects who finished at least 2-year follow-up comparing the situation at baseline and at the end of the follow-up. *ACEI*, angiotensin-converting enzyme inhibitor; *ARB*, angiotensin II receptor blocker; *MRA*, mineralocorticoid receptor antagonist

### Procedure and Safety Aspects

On average, 2.9 anchor pairs were used for reconstruction, of which 1.3 anchor pairs were implanted as LV-LV. The mean total operating time was 204 ± 50 min, and the mean time for implanting the anchoring system was 77 ± 34 min.

The anchoring system was successfully implanted in 22 patients (96%). In one patient, conversion to sternotomy was indicated due to acute mitral regurgitation caused by chordae impairment. Two patients were subjected to conventional re-operation after 3 days and 6 weeks after LV reconstruction, respectively, due to deteriorating tricuspid valve insufficiency. Even at the rest of patients, we observed some tricuspid valve insufficiency progression compared to baseline grade 0.64 ± 0.6 (scale 0–4): 1.68 ± 0.8 after 6 months (*p* < 0.001), 1.18 ± 0.8 after 2 years (*p* = 0.08), and 1.65 ± 1.0 after 5 years (*p* = 0.003). Four patients died before their 5-year follow-up visit: the first patient died 2 weeks after the procedure due to combined shock with multiple organ failure and acute abdomen, the second 4 months after intervention as a consequence of periprocedural ischemic stroke, the third 8 months after the procedure due to lung carcinoma, and the fourth 4 years after the procedure as a result of alcohol abuse. This resulted in all-cause mortality rates 4% at 30 days, 13% at 2 years, and 24% at 5 years.

### Effect on Left Ventricular Remodeling

**Fig. 1:**
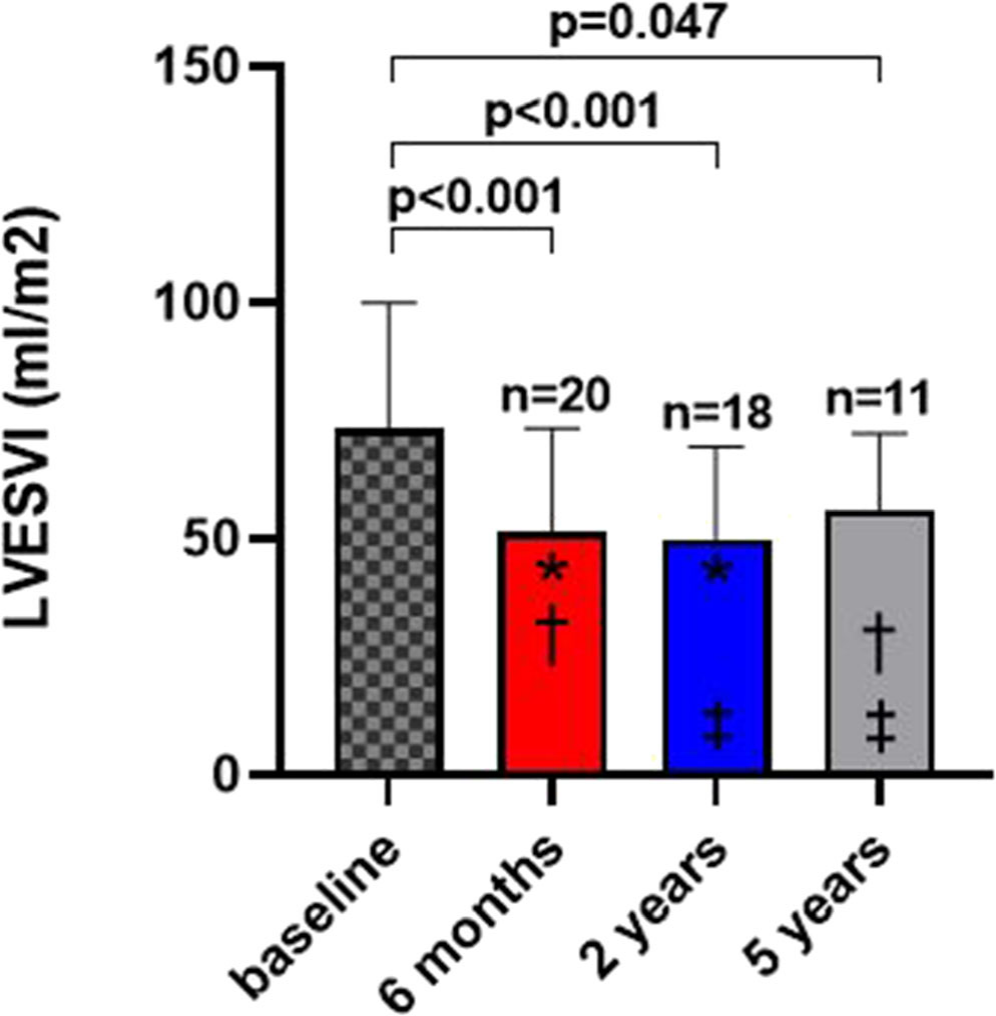
Change in LVESVI at 6 months, 2 years, and 5 years. Data are expressed as mean ± standard deviation. *p = ns, †*p* = ns, ‡*p* = ns. LVESVI, left ventricular end-systolic volume index

**Fig. 2:**
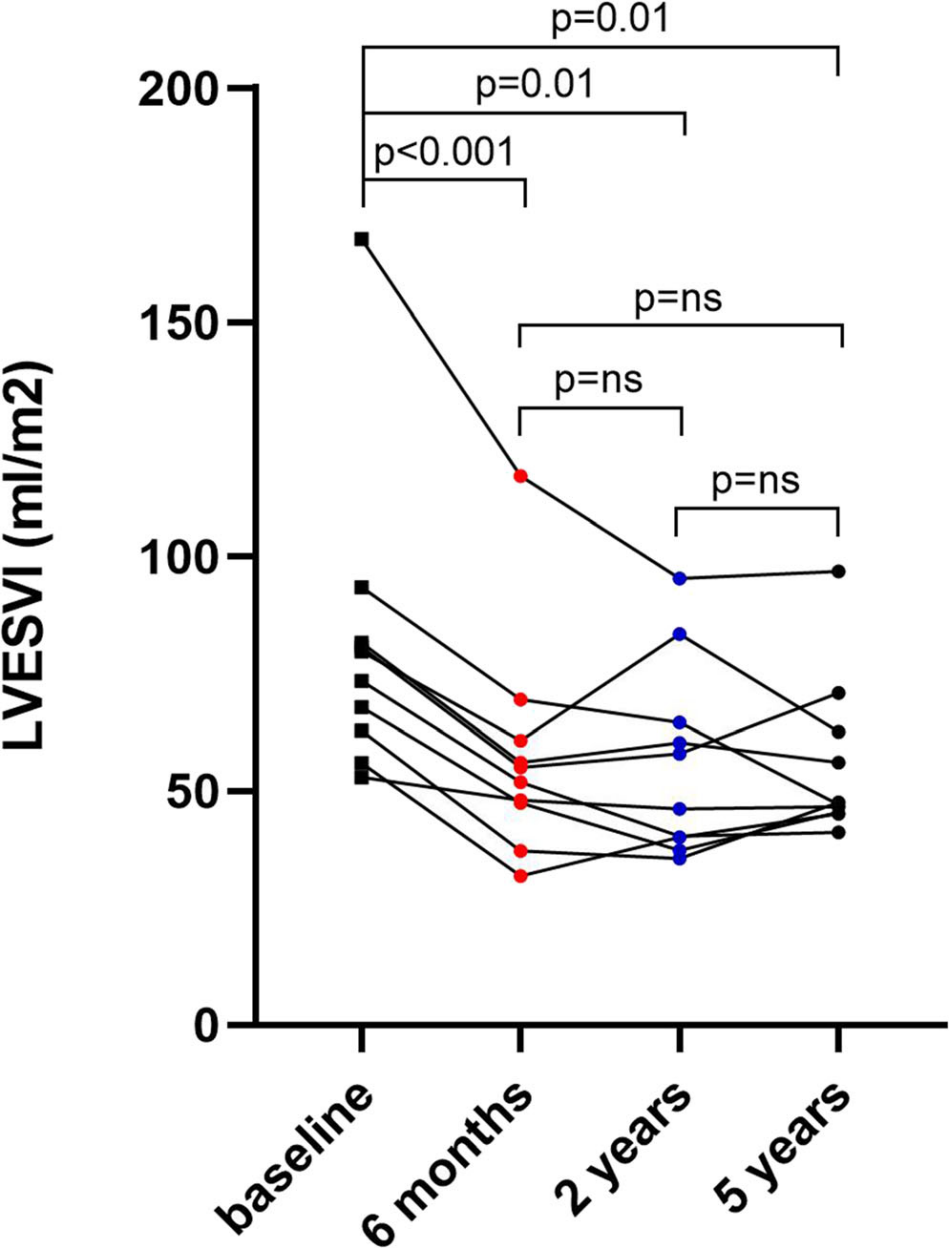
Individual changes in LVESVI at 6 months, 2 years, and 5 years. LVESVI, left ventricular end-systolic volume index

**Fig. 3:**
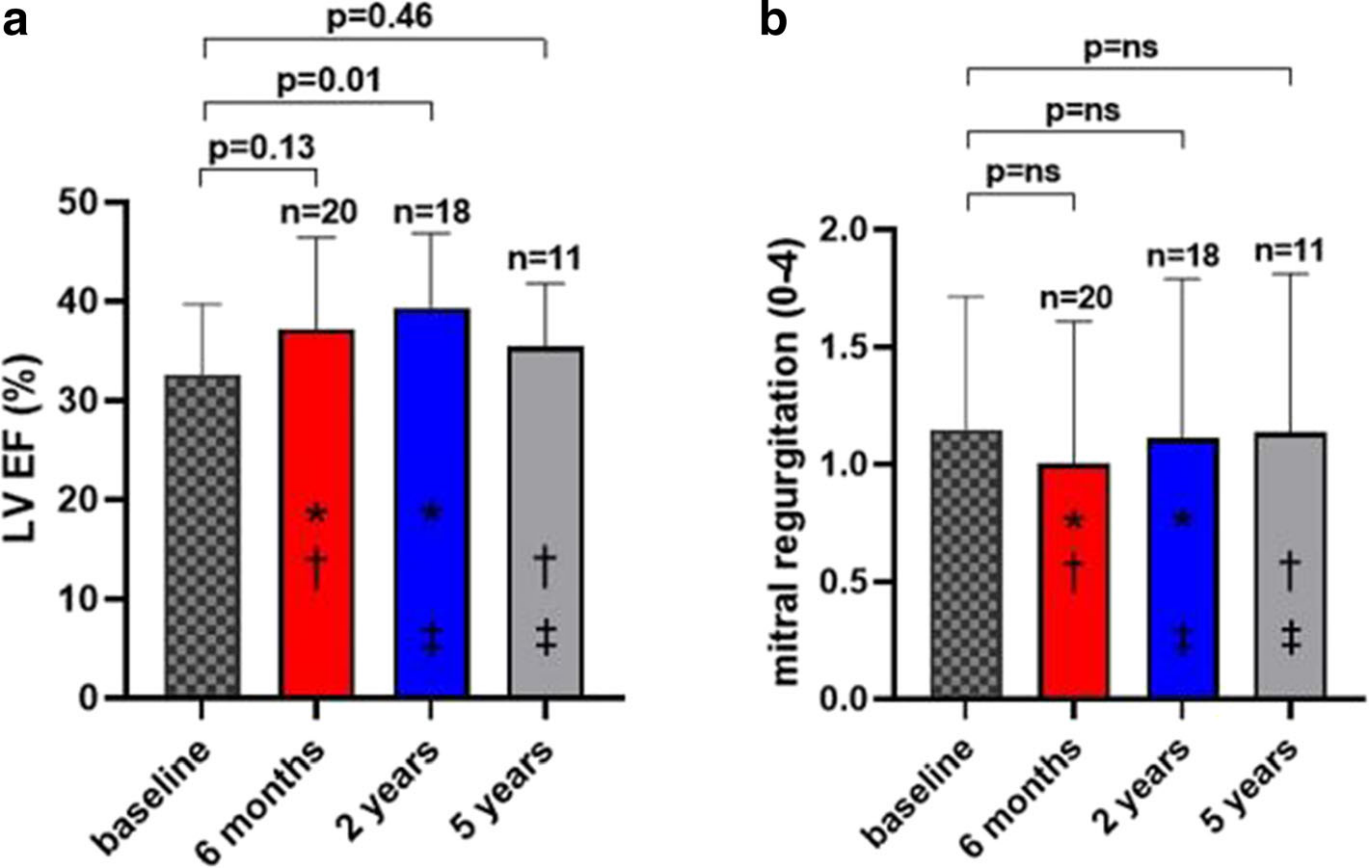
Change in left ventricular ejection fraction (**a**) and mitral regurgitation (**b**) at 6 months, 2 years, and 5 years. Data are expressed as mean ± standard deviation. *p = ns, †*p* = ns, ‡*p* = ns. LV, left ventricle; EF, ejection fraction

As shown in Fig. 1, LVESVI was significantly reduced from 73.2 ± 27 ml at baseline to 51.5 ± 22 ml after 6 months (*p* < 0.001), 49.9 ± 20 ml after 2 years (*p* < 0.001), and 56.1 ± 16 ml after 5 years (*p* = 0.047). LVESVI reduction persisted throughout the 5-year follow-up period in individual subjects (Fig. 2). The relative LVESVI reduction after 6 months, 2 years, and 5 years was 30%, 33%, and 31%, respectively. LV end-diastolic volume index significantly decreased (Supplemental Figure 1) and the LV EF increased, though significantly only at 2 years (Fig. 3a). Mitral regurgitation was not affected by the procedure (Fig. 3b).

### Effect on Symptoms, Functional Capacity, and NT-proBNP Level

**Fig. 4:**
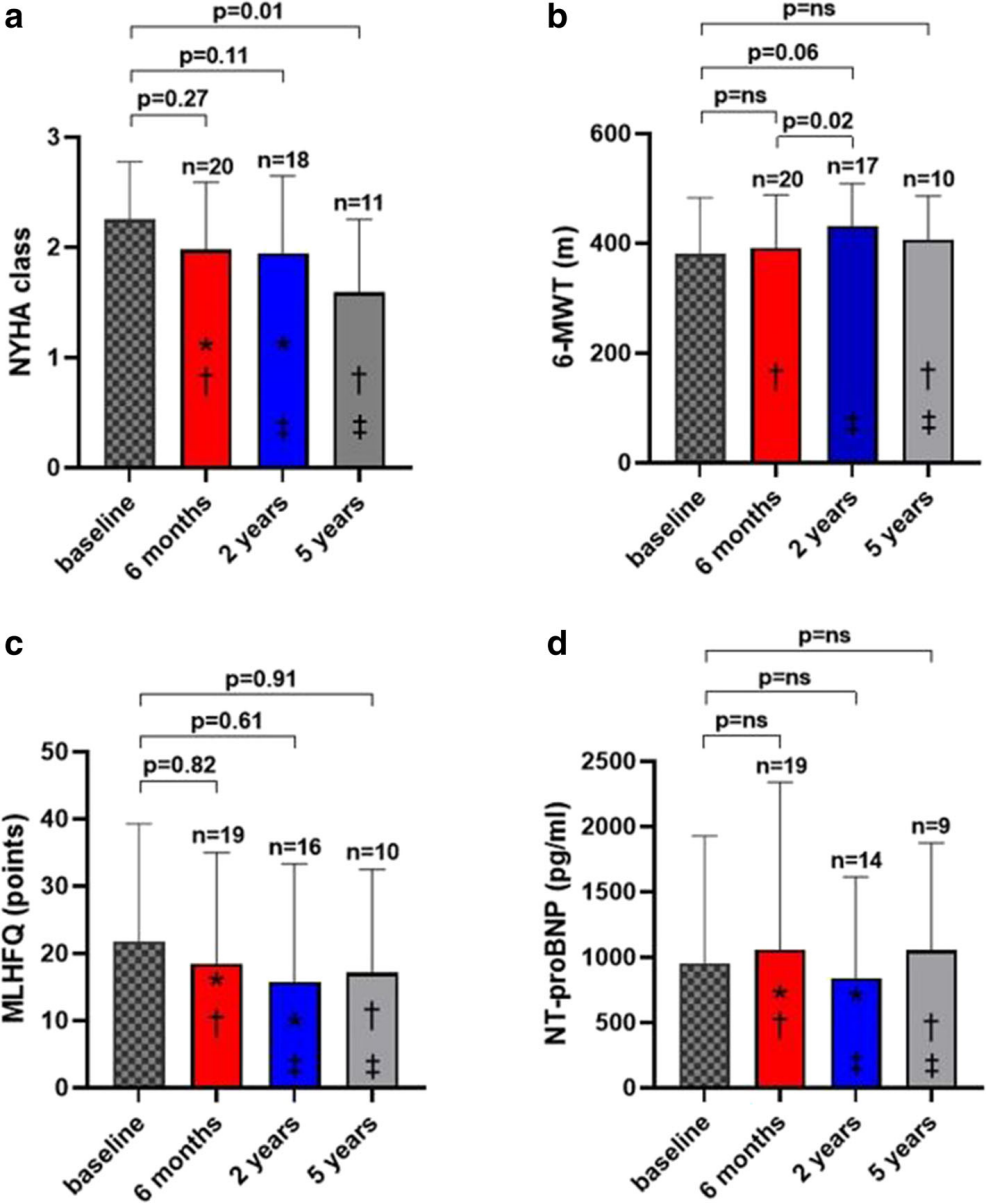
Effect on clinical parameters relevant to HF and NT-proBNP at 6 months, 2 years, and 5 years. Data are expressed as mean ± standard deviation. *p = ns, †*p* = ns, ‡*p* = ns. MLHFQ, Minnesota Living with Heart Failure Questionnaire; NT-proBNP, N-terminal prohormone of brain natriuretic peptide; NYHA, New York Heart Association; 6-MWT, 6-min walk test

NYHA class decreased significantly after 5 years compared to baseline (2.3 ± 0.5 versus 1.6 ± 0.7, *p* = 0.01) — Fig. 4a. There was a significant improvement in 6-MWT at 2 years compared to the 6-month visit (392 ± 97 versus 432 ± 77 m, *p* = 0.02) and trend to the improvement at 2 years compared to baseline — Fig. 4b. Changes in MLHFQ and NT-proBNP levels were not significant (Fig. 4c–d).

### Effect of Baseline LVESVI on Clinical Parameters Relevant to Heart Failure

**Fig. 5:**
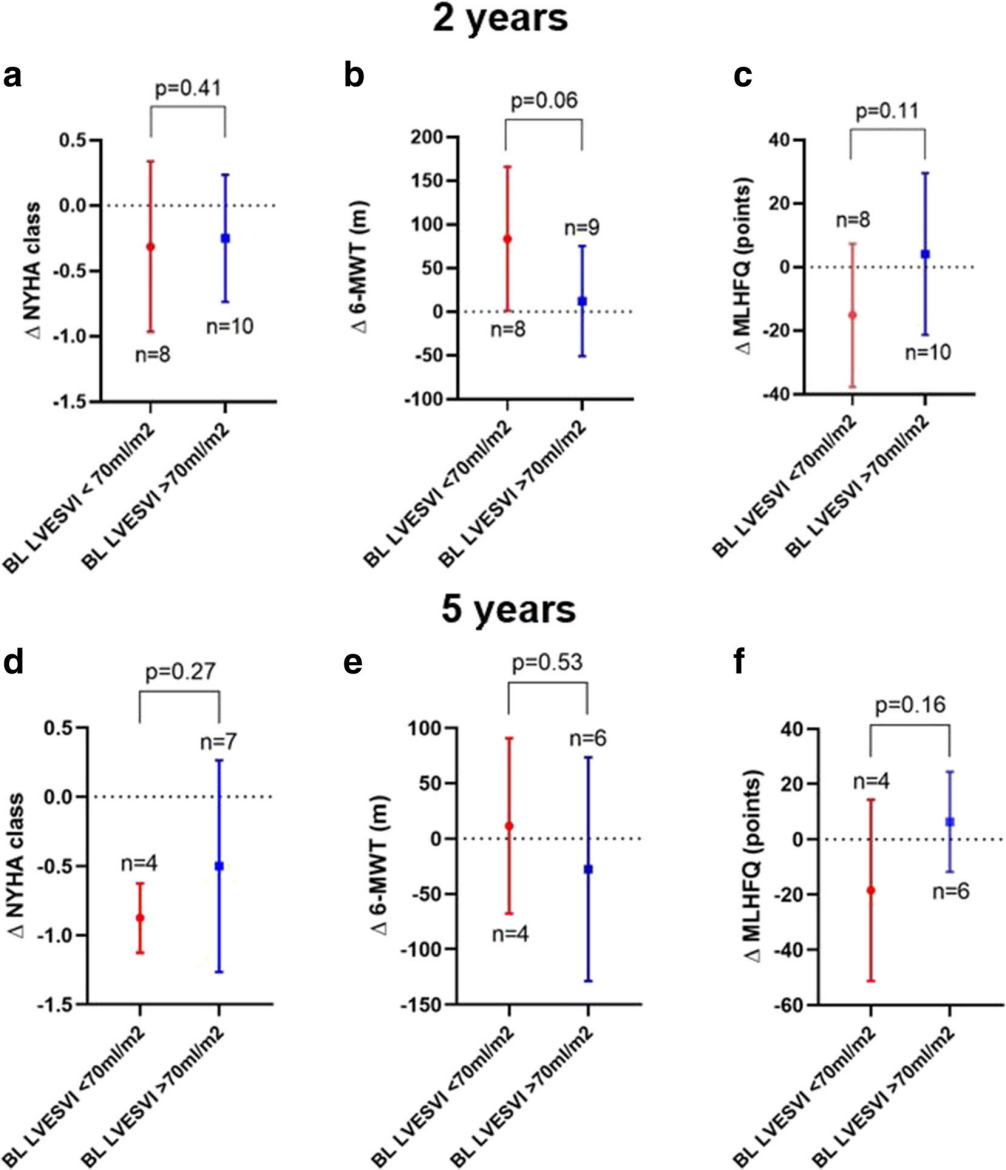
Effect of baseline LVESVI on clinical parameters at 2 and 5 years. Subgroup analysis based on preoperative LVESVI (cut-off 70 ml/m^2^) showing the effect of index procedure on NYHA (**a**, **d**), 6-MWT (**b**, **e**), and MLHFQ (**c**, **f**) at 2 and 5 years compared to baseline. Data are expressed as mean ± standard deviation of the change. *p = ns, †*p* = ns, ‡*p* = ns. BL, baseline; LVESVI, left ventricular end-systolic volume index; MLHFQ, Minnesota Living with Herat Failure Questionnaire; NYHA, New York Heart Association; 6-MWT, 6-min walk test

Figure 5 suggests that patients with smaller baseline LVESVI may benefit more from the procedure, but the differences did not reach statistical significance.

### Effect on Hospitalization Rate

Supplemental Table 1 shows number of unscheduled all-cause and HF hospitalizations comparing 2- or 5-year follow-up period with equivalent 2- or 5-year period prior index procedure.

## Discussion

In present study, we have shown that hybrid transcatheter and minithoracotomy LV reconstruction with the Revivent TC anchoring system is a feasible method enabling significant and durable LV volume reduction (relative LVESVI reduction 30–33%). LV reverse remodeling effect was accompanied by improvement in NYHA class and 6-MWT in certain parts of the follow-up; however, significant changes in MLHFQ or NT-proBNP were not detected. An additional sub-analysis testing the role of baseline LVESVI on clinical outcome showed a trend towards improvement in all variables, but it did not reach statistical significance.

Regarding surgical LV reconstruction, preliminary data from registries and one small prospective randomized study demonstrated favorable outcomes in surgical LV aneurysmectomy, generally with concomitant coronary artery bypass grafting (CABG), on ventricular volume reduction, functional status, ischemic mitral regurgitation, and HF hospitalization burden [8–11]. Aguiar Ribeiro et al. demonstrated the benefit of LV reconstruction in addition to CABG in patients with ischemic cardiomyopathy in a small prospective randomized controlled single-center trial (*n* = 74) [11]. NYHA class significantly improved and the incidence of HF recurrences and re-hospitalizations was lower in the LV reconstruction arm. Relative LVESVI reduction in the interventional group was *32%*. The largest randomized clinical trial (STICH), which compared CABG versus CABG plus surgical LV aneurysmectomy, failed to prove any benefit of LV reconstruction [12]. However, significant volume reduction was not achieved in the interventional group (relative LVESVI reduction was only *13%*). Post hoc subgroup analyses implied that patients with smaller baseline LV (LVESVI < 60 ml/m^2^) and better LV EF (≥ 33%) or patients that achieved postoperative LVESVI (< 60–70 ml/m^2^) may benefit from aneurysmectomy [16, 17]. Following the publication of neutral STICH trial results, data showing the beneficial effect of surgical aneurysmectomy *with adequate LV volume reduction* have been reported [19, 20]. Witowski et al. and Skelley et al. demonstrated, in single-arm studies (*n* = 79 and 87, respectively), significant improvement in symptoms or functional capacity after surgical LV reconstruction performed as a concomitant procedure, combined mostly with CABG. Relative LVESVI reduction was *41%* and *31%*, respectively [21, 22]. Likewise, higher baseline and post-procedure LVESVI signaled adverse outcomes. Recently, Klein et al. presented a multicenter trial with 12-month data after LV reconstruction using the Revivent anchoring system (*n* = 89) [14], of which only 41% of the patients (*n* = 35) were subjected to a hybrid minimally invasive procedure without sternotomy and concomitant CABG. The study showed statistically significant LV volume reductions, increased EF, and clinical and functional improvement. Low preoperative LVESVI seemed predictive of positive response to remodeling therapy. Adequate volume reduction and early clinical improvement with the Revivent TC system was reported also by Wang et al. from a single-center series of 26 patients [15].

In the present study, the short-term changes of LVESVI were very similar compared to 1-year data in Klein et al.’s trial. However, the main finding of the present study with potential clinical implication is that the significant volume reduction induced by index procedure persisted throughout the 5-year follow-up. Statistical non-significance of the majority of clinical parameters may be explained partially by the small sample size but may also be due to the less symptomatic cohort enrolled in our study compared to Klein et al. (baseline NYHA 2.3 ± 0.5 versus 2.6 ± 0.5, 6-MWT 381 ± 103 m versus 345 ± 108 m, MLHFQ 22 versus 42 points). Two- and 5-year all-cause mortality of the patients who underwent LV reconstruction in the STICH trial was approximately 50% higher than in our study. Nevertheless, this comparison is arguable for many reasons — different sample sizes, enrollment period difference more than a decade, or concomitant CABG in STICH trial.

### Limitations

The present study has several limitations. First limitation is a non-randomized uncontrolled design of the trial. Lack of control group makes the interpretation of changes in NYHA class, MLHFQ, or exercise capacity after 2 or 5 years problematic. It is highly disputable whether the observed improvement in 6-MWT at 2 years and NYHA class at 5 years is in relation with the index intervention. Propensity matching with the patients receiving optimal medical therapy could be helpful. There might also be some influence of selection bias on observed variables due to mortality of patients during follow-up, in terms that only patients with more favorable outcome were finally included in the analysis. Similarly, interpretation of pre- and postoperative hospitalization rate is highly disputable in absence of control group, since HF with reduced EF is characterized by progressive character. Second limitation represents the small sample size that limits especially the statistical analysis of secondary endpoint variables at 5 years and proper subgroup analysis. Nonetheless, despite the fact that the interpretation of secondary endpoints, unscheduled hospitalizations, and subgroup analysis is disputable, to date, this is the largest presented cohort of patients intervened using the Revivent TC system with > 1-year follow-up and the effect on LV remodeling was clearly proven even with this sample size. Third limitation represents the echocardiographic evaluation of LV volumes. Echo data analysis may be impaired by artifacts after anchor implantation. Volume calculation was not done by multiple readers. Fourth limitation is that the background medical therapy of HF did not remain unchanged during the follow-up (dose titration, new initiation of sacubitril/valsartan in three patients) that may also have an influence on patient outcome.

## Conclusion and Prospects

Hybrid transcatheter and minithoracotomy LV reconstruction using the Revivent TC anchoring system appears to be a feasible treatment method achieving substantial and durable left ventricular volume reduction. It offers the advantage of minimally invasive procedure without need of sternotomy and cardiopulmonary bypass. Long-term effect on clinical parameters and mortality needs to be confirmed in a larger randomized controlled clinical trial.

## Supplementary Information

ESM 1 **Video 1a-d**. *Title:* Representative example of two-dimensional echocardiography of one subject. *Legend:* Left ventricle is displayed from apical four-chamber view preoperatively **(a)**, at 6 months **(b)**, 2 years **(c)** and 5 years **(d)** after reconstruction. On postoperative images, the shadow of the anchor is visible on the apical part of interventricular septum (MP4 10856 kb)

ESM 2 (MP4 5782 kb)

ESM 3 (MP4 7853 kb)

ESM 4 (MP4 6952 kb)

ESM 5 (JPG 28 kb)

ESM 6 (DOCX 12 kb)

## Abbreviations

CABG: Coronary artery bypass grafting
EF: Ejection fraction
HF: Heart failure
LV: Left ventricle
LVESVI: Left ventricular end-systolic volume index
MLHFQ: Minnesota Living with Heart Failure Questionnaire
NT-proBNP: N-terminal prohormone of brain natriuretic peptide
NYHA: New York Heart Association
6-MWT: 6-min walk test
