# Supplementary figures and images for: Long-Term Results of Hybrid Left Ventricular Reconstruction in the Treatment of Ischemic Cardiomyopathy
Source: J Cardiovasc Transl Res. 2021 May 11;14(6):1043–50. doi: 10.1007/s12265-021-10133-9 (PMC8651588; doi:10.1007/s12265-021-10133-9)

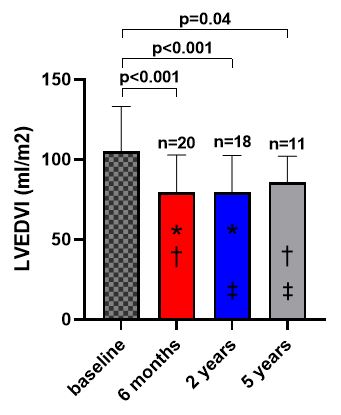

Supplement: Supplementary file 5 — (JPG 28 kb) [file 12265_2021_10133_MOESM5_ESM.jpg]
