# Supplementary material for: Long-Term Results of Hybrid Left Ventricular Reconstruction in the Treatment of Ischemic Cardiomyopathy
Source: J Cardiovasc Transl Res. 2021 May 11;14(6):1043–50. doi: 10.1007/s12265-021-10133-9 (PMC8651588; doi:10.1007/s12265-021-10133-9)
Supplement: Supplementary file 6 — (DOCX 12 kb) [file 12265_2021_10133_MOESM6_ESM.docx]

**Supplemental Table 1**

*Title:* Hospitalization rate.

|  | Preoperatively  (n = 19) | Postoperatively  (n = 19) | P-value |
| --- | --- | --- | --- |
| All-cause hospitalizations | 19 | 19 | 1 |
| Heart failure hospitalizations | 5 | 7 | 0.42 |

Number of unscheduled hospitalizations comparing 2-year (n = 8) or 5-year (n = 11) follow-up period with equivalent 2-year or 5-year period prior index procedure.
